# A novel methodology of international discourse: online joint course across cultures

**DOI:** 10.1057/s41599-022-01233-1

**Published:** 2022-06-25

**Authors:** Zsuzsanna Schnell, Christopher Podeschi

**Affiliations:** 1 University of Pécs, Pécs, Hungary; 2 Bloomsburg University of Pennsylvania, Bloomsburg, USA

**Keywords:** Education, Cultural and media studies

## Abstract

This article explores a novel methodology in the globalized classroom to face and tackle new challenges across cultures. The new online educational form of international discourse has the potential to make a difference in and outside Academia as it encompasses interpersonal communication across cultures and thus offers a novel framework for interpretation of different sociocultural perspectives to bring about social change. It transcends geographical and physical boundaries as it brings people together for discussion in the online joint event. Our study guides the reader through a collaborative educational framework and offers a methodology that transcends boundaries, exemplifying a tool for 21st century education. It offers techniques that bring on social change and enhances open-mindedness in tackling social problems with valuable methods from the fields of humanities and intercultural, collaborative communication.

## Introduction

The present article investigates a novel approach in academic life and higher education, namely, collaborative international learning in an online, joint course framework; the realization of the course at the University of Pécs, Hungary, and Bloomsburg University of Pennsylvania, USA. Our joint course experience offers a new perspective in highlighting the psycho-social aspects of identity development; therefore, it represents a powerful educational tool for the generations of the twenty-first century. The intercultural discourse in the online joint course setting is unique in the sense that it is possible for a number of different cultures to meet allowing participants to gain insight into the topic at hand from very different cultural and social perspectives. This platform ensures that participants become more open minded, which acts as a facilitator of psychosocial development of identity. Their own beliefs and opinions on the topic receive instantaneous and reflective opinions from very different cultural perspectives. This enables the integration of differences at the cultural, social, linguistic and psychological level, empowering education with extremely effective tools for intercultural discourse and creating the possibility of social change.

Key challenges of the 21st century include many current issues like the social effects of migration, cultural and psychosocial consequences of globalization, or the question of gender issues in different societies. The academic discourse our joint course framework offers is one that can make a difference inside and outside the Academia by enabling students to experience cultures in an immersive way, experience the integration of different cultural perspectives at the personal level, and see the harmonization or the clash of different cultural values.

## Background

The societal challenges of modern cultures include problems of globalized and localized cultures, psychological and social aspects of identity formation and issues of locality and identity. Overcoming in-group/out-group tension, even in its milder forms, is of crucial importance. We believe that to make a difference in and outside the Academia, education of the twenty-first century must be a pedagogy aiming at social change. Pedagogy for change must actively involve participants in the process of learning. Very different from traditional approaches of frontal instruction style education, today’s students are not willing to be passive and silent note-takers. They need to be active and inquisitive participants in the process of scientific communication (Mackenzie-Bathurst-Hunt, 2018).

The task of educators goes beyond just choosing a subject, getting a degree and working in a school. Being an educator goes together with changing the education system and adapting it to the needs of the generations of the 21st century. The reason why we believe collaborative online academic discourse can make a difference inside and outside of Academia, and that education is an efficient tool to bring about social change, is the fact that one of life’s greatest achievements is to inspire other people, and to teach them of lessons learned and conclusions drawn from first-hand personal experience. This is rarely possible in the traditional setting where students mostly discuss problems in their own cultural bubble, not getting different cultural perspectives. The old ways of education need to be complemented with new approaches that are relevant for the students of this century. Education that enables intercultural discussion of topics helps to open minds. We believe education serves as a powerful tool for change. We offer an account of our joint course approach and with that, a new perspective on social change for our new generations, with a qualitative method with the aim of offering solutions from approaches widely present in the Academia from the fields of humanities and social- communication studies.

## Basics of the Collaborative Online International Learning (COIL) methodology

In the present study we share the experience of designing and executing an online, joint-course where the University of Pécs, Hungary and Bloomsburg University of Pennsylvania, USA collaborated to investigate sociolinguistic- and psycholinguistic aspects of social identity and intercultural communication in an interdisciplinary framework. The approach generally followed the “Collaborative Online International Learning” (COIL) framework (see SUNY Coil Center, n.d.). In our course the instructors take turns giving interactive lectures while classes follow on two different continents, with several different cultures represented. The students themselves often represented several strikingly different sociocultural backgrounds ranging from the Far East, the Middle East, Central Europe, and the United States.

### Unique aspects of the online joint course methodology

This online methodology is not only a unique example of the now predominant “Zoom” courses widely used due to Covid 19 but is also one that supports the development of twenty-first century skills in education using a computerized platform. In the era of smart-phones and technologically savvy students immersed in social media, there is strong need for genuine human connection, wisdom and commonsense thinking, together with opportunities to develop interpersonal abilities of social-sensitivity that integrate psychological and cognitive emotional factors. The wisdom of first-hand, experience-based conclusions and the mind-opening effects of being well-traveled are both unique aspects of the course that participants are given, even though they do not necessarily have to leave their classroom for intercultural experience at all. This platform offers a unique, applied aspect of discussion, as reflections on the topic at hand are discussed from very different perspectives, yielding incredibly rich intercultural interpretations of problems. Frameworks of interpretation were very much widened by the variety of cultural backgrounds of the students participating, where the individualist perspectives of Americans were often confronted with interpretations of collectivistic cultures in the Middle- or Far-East, and with those of Central Europeans in the middle of the spectrum, each with perspectives that shed light on the impact of linguistic and cultural relativity of interpretation, like in the case of the dress code and its social corollaries, or leaders’ effect on one’s national identity.

### Academic discourse with novel methodology for educators

In the novel joint course, context is of key importance, as it offers an applied aspect of academic discourse. Participants themselves are representatives of their local culture, and these individual perspectives get incorporated in the wider communicative matrix and paradigm of the course. This incorporates both the individual, personal aspect and the community-level aspects as well in the reflections. These two levels of communication give further, prism-like reflections, in that they multiply perspectives, as there is a continuous discussion of different opinions, angles and contexts generating novel frameworks of interpretation.

**Fig. 1:**
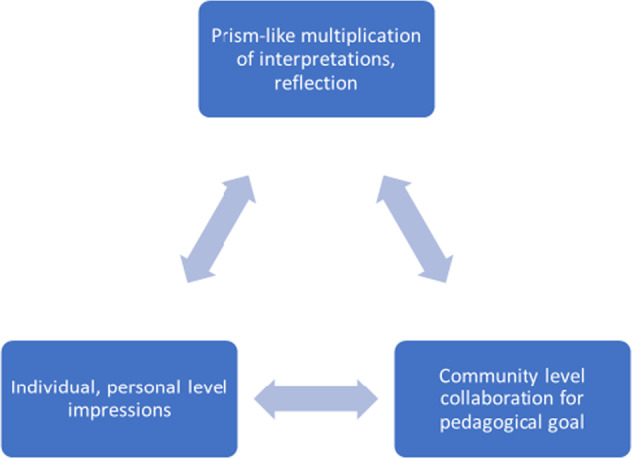
The multiple levels of academic discourse in the collaborative online methodology.

In such a framework different cultural adaptations and applications are revealed, generating novel perspectives and frames of reference for participants to see other cultural interpretations. Such implications resulting from the prism effect of multiplied cultural perspectives articulated during academic discussion eventually impact the perspectives of participants. This in turn, enables interpretations of the topic at hand at applied levels, where individuals act as representatives of cultures and their understanding of the problem gets instant and genuine reflections, opinions incorporating different angles, that in turn receive responses. Online activities are of key importance in this method, where the actual responses, during pair work and teamwork, allows the digestion of different impressions. The platform is therefore rich in multifaceted cultural, real first-hand experience with realistic input (Fig. 1).

### New challenges in 21st century education

**Fig. 2:**
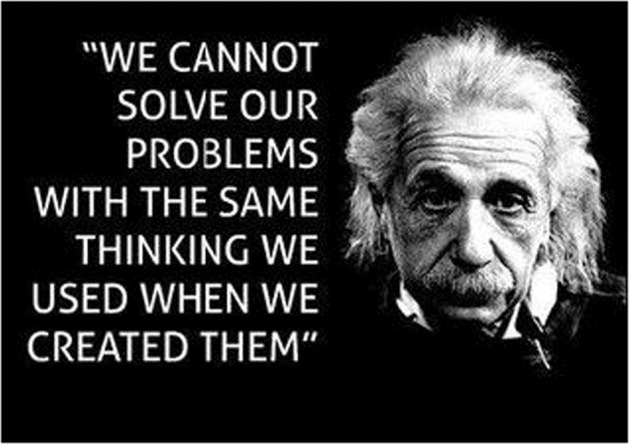
Meme about the need for new perspectives for social change. source: https://quotesgram.com/einstein-problems-quotes/.

To tackle the challenges of the 21st century we need novel tools. A famous saying, often attributed to Albert Einstein, tells us that we cannot solve our problems with the same thinking we used when we created them (Fig. 2). Novel methodologies offer new perspectives and solutions, new forms of *communication* and *collaboration* where *creativity* and *critical thinking* can shed new light on ordinary ways of functioning.

**Fig. 3:**
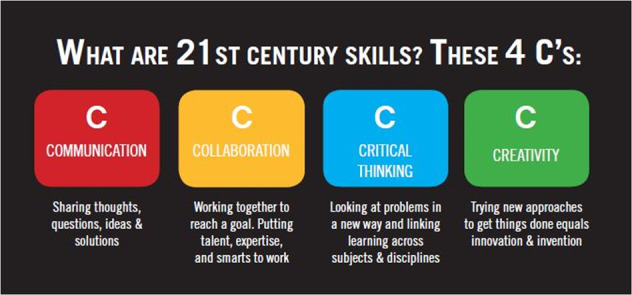
The 4C’s in 21st century skills (Schnell-Zalay-Gombás, 2021).

These 4Cs are well-known to be the basic pillars of 21st century skills (Fig. 3). Amid problems like global migration that triggers intercultural conflicts, humane approaches stemming from the ability to understand others’ minds, accept differences, and respect diverse values all converge in the direction of the need for skills people in media-saturated high-tech societies lack.

In the process of technological development, we gradually get more and more distant from our original root culture and common-sense knowledge we called wisdom, where humane communicative abilities, social cognition with which we interpret others’ minds and predict their intentions, is crucial. Such skills are indispensable for students of the twenty-first century in managing novel problems in society at local and global scales. Instant solutions generated by the mass-media and smart technologies are in fact eroding mindfulness and the abilities to contemplate and understand others. Such new circumstances call for new solutions and the harmonization of views across cultures and continents. Academia, a milieu of the educated, is a perfect platform for that, and discourse in this realm has the power of bringing on social change by reinforcing the 4Cs, the basic competencies twenty-first century modern humans need most. Integrating these in education, bringing them into discourse in and outside of Academia, has the potential to transform societies and basic cognitive schemas today.

### Academic discourse for social change: Educators gain a new role

In the academic discourse of the twenty-first century teachers’ roles must change; rather than having traditional of “all-or-nothing” roles, or being a source of all basic knowledge, they must be mediators. They must embrace the basic knowledge the students already have, as they have had their smart devices throughout their development and have thus spent years “Googling” numerous specific topics. It is hard for educators to keep up with the instantaneous, multi-modal, quickly changing, colorful, music-driven communication that smart devices offer. However new generations are also overwhelmed with communicative stimuli, and this arguably means they no longer belong to genuine communities. They get the false image of belonging to many communities, but these are virtual and based on weak or inauthentic connections (Szécsi, 2013, 2021).

**Fig. 4:**
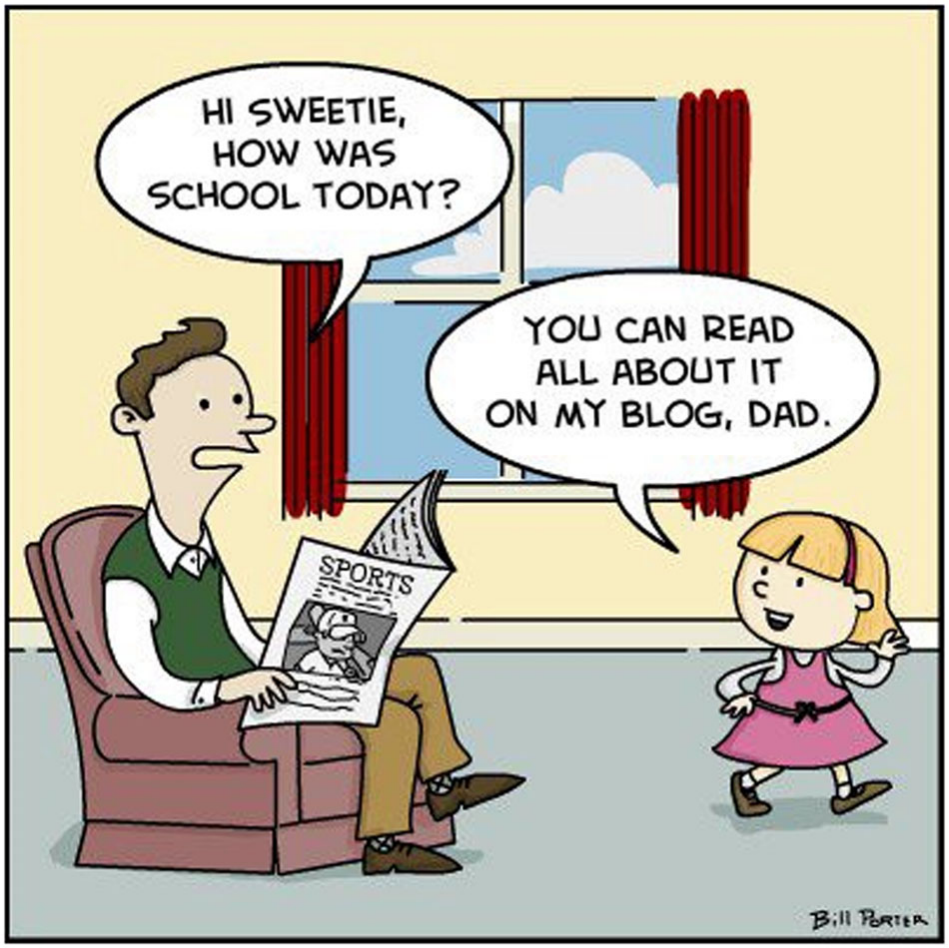
The generation of digital natives pose new challenges in academic discourse source: https://medium.com/digital-reflections/digital-natives-and-digital-immigrants-how-are-they-different-e849b0a8a1d3.

Educators today are not there to teach basic skills, as the *X*, *Y*, and *Z* generations often have a better grasp of the new technology skills than their teachers also known as digital immigrants, trying to teach digital natives (Schnell-Zalay-Gombás, 2021). Educators need to build on the skills these students have developed for themselves (Fig. 4). The integration of their personal skills and individual strengths and interests reinforces the learning process as it strengthens the individual level in the academic discourse. This in turn strengthens their group identity and facilitates their involvement and participation in the discussion of the topic at hand.

**Fig. 5:**
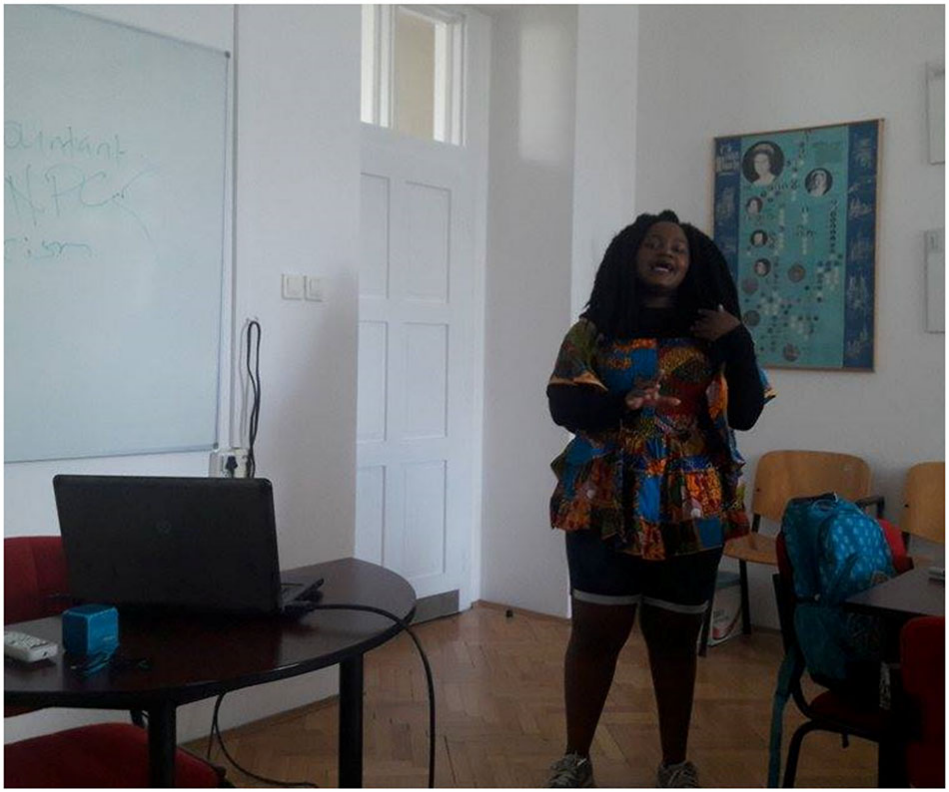
Our student Nora from Nigeria giving a presentation about her culture in their traditional clothing she brought for this special occasion.

Some students in today’s classrooms will work in jobs that don’t yet exist, hence social-cognitive competencies, mindfulness, and skillful understanding of the beliefs, intentions, and desires of others in order to predict and understand their behavior is crucial. These are the human-specific abilities no machine can do as well as humans (Schnell, 2015). This is the most important challenge of Artificial Intelligence (AI) today (Pléh, 2000, Schnell, 2016, 2019). Such social-cognitive competencies will increase the chances of successfully navigating the challenges of twenty-first century societies. These abilities are crucial for adapting to new circumstances in this continually changing globalized, migration- and pandemic-impacted world. We all need to adopt new perspectives for harmonious interaction with other cultures, and to understand the minds of others in social relationships to limit or reverse the alienation fostered by virtual communities and virtual connections. Such methodologies can also enable us to flexibly apply knowledge and skills in novel situations. Intercultural communication, therefore, offers important skills that bring on social change through academic discourse. (Fig. 5).

### The crucial role of technology in globalized online academic discourse

**Fig. 6:**
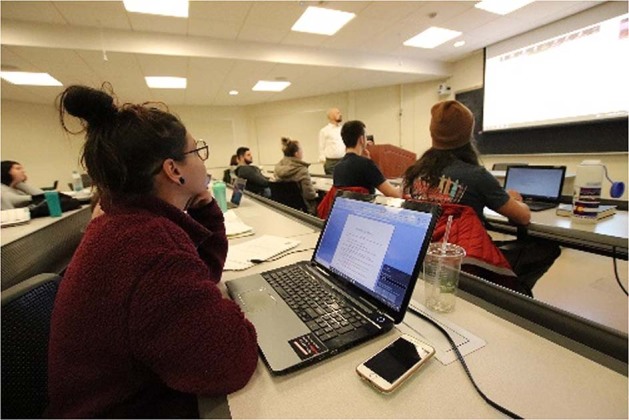
Joint course setting: partner university’s class projected on the large screen, sharing genuine experience via virtual platform.

New methods build on new tools. In our novel joint course computers are essential (Fig. 6), especially in social sciences, as they ensure an online platform, with a myriad of real-life videos that provide real linguistic and cultural input and stimuli. The internet has seemingly infinite resources; it is there for all players in Academia as a goldmine of teaching resources. It offers multimodal materials for all types of learners: auditory, visual, cognitive, kinesthetic, and imitative (Schnell-Zalay-Gombás, 2021; Schnell-Fóti, 2021). The internet even provides immediate first-hand experience from different cultures and perspectives, as it enables the realization of the joint course with real-time impressions, reflections, and intercultural discussions.

### Joint-course: key for 21st century competencies

The joint course paradigm enables students to encounter different cultures in real-time. Opinions formulated can be shared online, giving first-hand experience with different cultures via face-to-face interaction, where question-answer based instant clarifications give genuine impressions of the target culture and population (Mackenzie-Bathurst-Hunt, 2018). The intercultural context is perfect for the understanding of specific topics that might have different cultural interpretations. This is especially valuable in social sciences where social phenomena are investigated in a culture-specific manner, through the researcher’s lenses. In the online joint course paradigm one can get a new perspective on the spot through question-and-answer sessions which can fight discrimination, xenophobia, and similar social problems. This novel platform is perfect for a collaborative framework for finding new solutions by discovering the common grounds and by bridging differences in a community of students and professors from various cultures.

### The course as an international collaboration

**Fig. 7:**
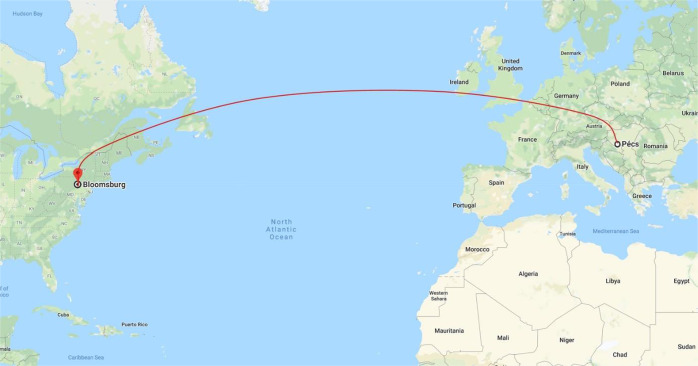
University of Pécs (UP)—Bloomsburg University of Pennsylvania (BU) collaboration bringing together various cultures and continents.

The course is a result of several years of collaboration between the University of Pécs and Bloomsburg University of Pennsylvania (Fig. 7), Targeting international-scale interdisciplinary research, the Grastyán Collegium for Advanced Studies at the University if Pécs provided support as well.

The course focused on topics in sociology, psychology, linguistics and intercultural communication. The course titles, “Language, Culture, and Identity” (at the University of Pécs), and “Identity and Locality” (at Bloomsburg University), themselves reflected interdisciplinarity and students from different fields could sign up for it and join. We targeted the psycho-social factors of personality and identity development, starting with Professor Podeschi exploring a sociological understanding of locality and identity. Professor Schnell then covered the effects of linguistic and cultural relativity, issues of psycho-social development and factors of identity development (based on Erikson’s theory). We eventually discussed problems of globalization, glocalization and crucial issues in locality and identity (Erikson, 1993): how our local spaces, and place relationships influence and shape our personality, identity and therefore our societies and communities.

### Outline of the course

**Fig. 8:**
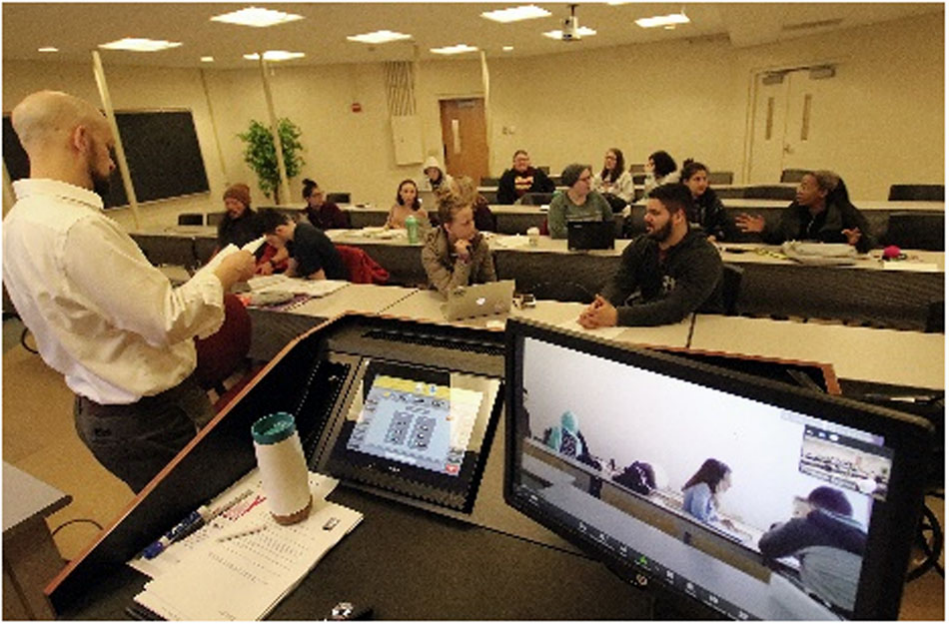
Professor Podeschi coordinating the BU classroom. Visible on the screen is the Hungarian classroom’s multicultural community of students discussing the task at hand in real-time on the other continent.

The first class focused on forming a community for learning for the entire semester and thus was devoted to getting to know each other by setting up a study-group on Facebook, where we shared pictures of real-life events in our introductory speeches. Apart from the American students and Professor we had multiple students from China, some of whom were from the Uygur community, an oppressed minority group. It was striking to see how the individuals managed to break away from cultural expectations and frameworks of thinking and managed to see each other as equal individuals, full-right members of the same course with equal chances and rights. Long-term friendships formed during the course, breaking down discriminative barriers within cultures, which in itself is exceptional in social settings and communities. The online joint course methodology facilitates the breaking down of such barriers and joining hands for a better future and mutual understanding and respect of everyone’s cultural values. A UP student from Tunisia helped the other students learn new details about Arab cultures that helped undermine views of such societies as homogenous with regard to gender equality. When we asked her about the headscarf she wore everyday, she relayed that in Tunisian culture, it can actually symbolize resistance and serve as an expression of female freedom. This countered stereotyped beliefs about Arab cultures. Of course, the cultural spectrum included Hungarians, with their central-European perspectives influenced by their history of socialist rule and oppression. The group prepared reading journals based on literature assigned by both professors, and these journals were discussed at the beginning of each class, before going into further relevant issues, like the relationship of language and thought, linguistic relativity (Whorf, 1941), then issues of the sociology of place and place attachment and locality (Podeschi and Howington, 2011) (Fig. 8). An important aspect targeted the understanding of the social and environmental factors of identity development from Erikson’s theory, focusing on psychosocial events shaping our personality. The course ended with a focus on locality and identity where we identified the 3R’s—Relationships, Rituals and Restrictions. A matrix was filled in by participants in culture-specific pairs or teams, and then we discussed how these were culturally different or similar. This shed light on our understanding of locality, our connection to our place of living and its impact on our personality and identity formation.

#### Course methodology

The course introduced students to *Intercultural Communication (ICC)* theories and to concepts relevant in International Relations (*IR*) centering on issues of *Identity* and *Locality*. The UP course objectives targeted the impact of language and culture on communication more, and students were required to examine *discourse*, analyze conversations and communication involving distinctive *cultural* features and artifacts, then identify and *evaluate* communication similarities and differences, contrasts, within co-cultures and certain sub-cultures. This was organized in the framework of a *field-trip* where students are required to carry out their *own research* based on independent project work and ideas. Students also *developed strategies* to improve and upgrade intercultural communication in settings to make discourse in interpersonal and intrapersonal dimensions more efficient.

#### Requirements

As a prerequisite to the UP course all students were required to have an at least B2 level English to be able to follow classes. For the completion of the course they were expected to complete assignments, contribute with their independent work, and participate in our intercultural discussions. To ensure active engagement, participation counted for 50% of the final grade.

#### Course objectives

The UP course incorporated the teleconference sessions with the Bloomsburg group. In the UP objectives emphasis was placed on socio-cultural and psychological issues of language and communication. Upon the completion of the course participants became *able to explain* the relationship between culture and communication, *name*, *identify* and *evaluate* different contextual variables that influence communication in intercultural settings, *analyze* and *interpret* the influence of types of communication and discourse strategies, *create* practices for improving intercultural communication, *understand* and evaluate the relationship between identity and locality, and communicate in an interpersonal-, social-, cognitive- and geographical setting. Most of these abilities targeted and stemmed from the 4Cs, Creativity, Communication, Collaboration and Critical thinking. The course therefore largely fulfilled the requirements of a novel type of educational methodology and academic discourse for the development of twenty-first century skills.

### Structure of the course and synopsis of the syllabus of the joint course with a turn-taking paradigm

The teleconference sessions were the joint sessions, while the US group conducted their own course that integrated the joint course events with more intercultural discussions.

**Fig. 9:**
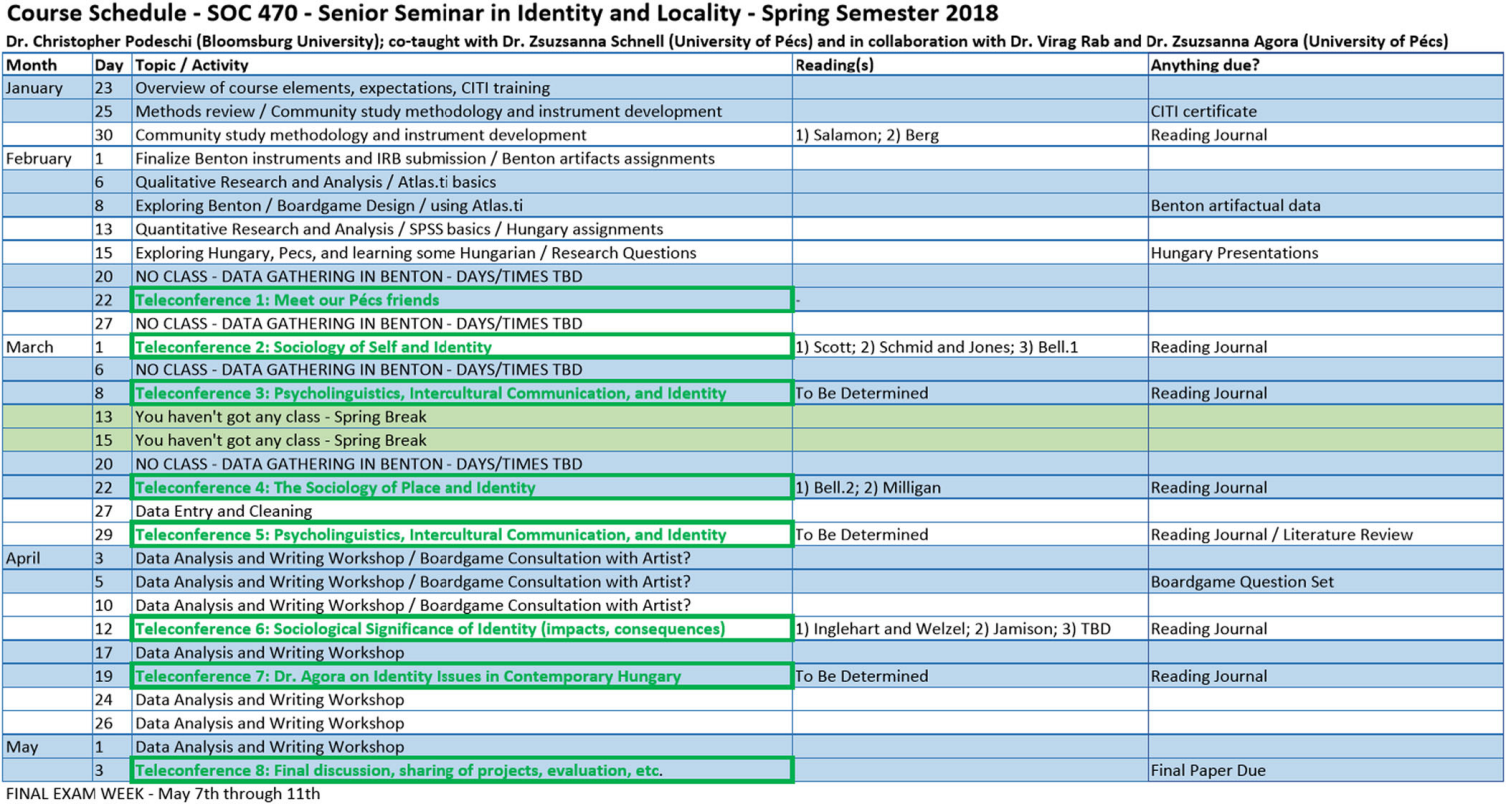
Schedule of the BU course, the turn-taking paradigm apparent with the joint-class sessions highlighted in green.

**Fig. 10:**
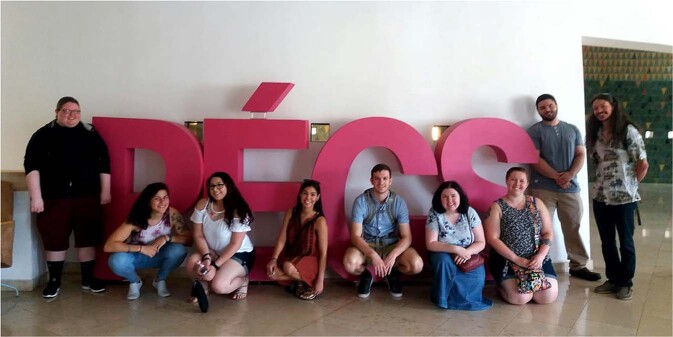
Visit of the Pennsylvania group to the University of Pécs for further learning.

There were eight teleconference sessions in all (Fig. 9). Organizing the course required a great deal of flexibility from both sides, from the professors and students alike, but also from both countries’ universities and staff. It required the harmonization of semester milestones, educational rules, finding the right place and time for the joint sessions—which was especially challenging as the two universities were in two different time zones. The professors took turns lecturing. Sessions typically included discussion of the students’ journal entries on the mutually assigned, harmonized obligatory literature. This was followed by a discussion of the given topic across cultures. The course and community building actually ended with the U.S. group’s visit in the city of Pécs, Hungary (Fig. 10). Nine of Professor Podeschi’s seventeen students were able to travel to Hungary and continue learning about culture and identity during numerous excursions, including to the town of Vajszló in rural Hungary, a community that paralleled the rural community in Pennsylvania that his students had studied.

### Applied aspects in the novel course methodology in academic discourse

The joint course focused on the uncovering of local values and extended the interpretation of their impact on identity development and thus community formation, transferring the topic of identity-locality research to the community level. The applied aspect of this scientific discourse focused on the target language and culture (to be mapped) vs. source culture and language (home culture). In the process participants discovered shared values and shared cultural frameworks despite what appeared to be significant cultural differences between Western, Middle Eastern, and Far Eastern cultures. We also used our online study group for reflections after every class and thus had a vivid exchange of ideas, impressions, experiences all throughout the week and semester with very personal insights.

## Collaborative stance to bring about social change

**Fig. 11:**
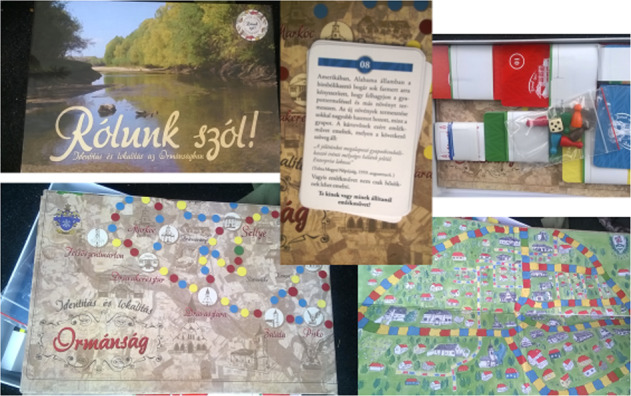
Contents and design of the board game to strengthen collaboration as a twenty-first century skill in academic discourse.

The University of Pécs previously took part in an innovative project and produced a board-game that targets interpersonal skills and locality-identity issues by strengthening the identity of people living in rural towns suffering population loss and changes. The board game developed by Professor Agora in the framework of the Grastyán College for Advanced Studies of the University of Pécs was used for community building and team-building purposes by the Bloomsburg group and by members of the Pécs group as well. The title of the board game is “It’s about us!” (“Rólunk szól!”), and it is based on the idea of enhancing personality development and identity development issues, with a subtle psychological methodology, incorporating knowledge of cooperative pedagogy. It is a game of collaboration, rather than competition in which collaboration brings success. Importantly we would like to highlight the applied nature of academic discourse in this project, as well. The integration of different cultural perspectives, this time not only in the inter-cultural but also in the intra-cultural dimension, gives the game its unique collaborative aspect. The cooperative stance as a core methodology here gained an applied, real physical aspect and was implemented into everyday educational practice, which makes this board game relevant and especially fruitful in the coil framework aiming to enhance collaboration between cultures in an intercultural online learning platform, where the respect of individual and local values are central. The contents of the board game can be seen in Fig. 11.

## Course outcomes and conclusions

**Fig. 12:**
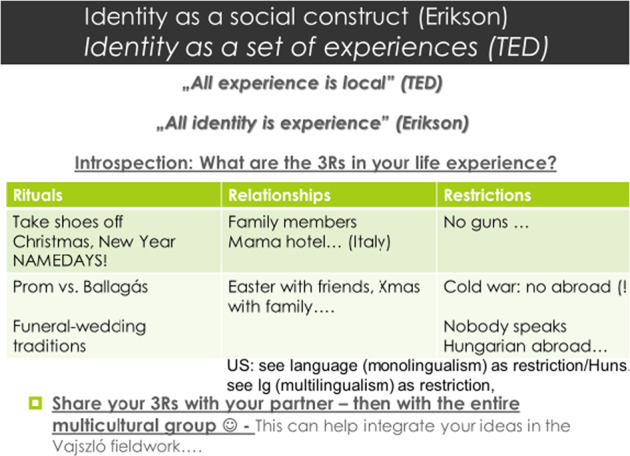
Matrix of three R’s for the understanding of the intertwined nature of Locality and Identity development.

The final session ended with students forming international pairs (one student from the U.S. group and one from the University of Pécs group that however included international students from several other cultures and countries, including the Far East and the Middle East, besides Europe). We deliberately created intercultural pairs in order to facilitate the discussion of cultural differences and divergent aspects of values. The pairs were required to create their own power-point presentation on the three R’s (Rituals, Relationships and Restrictions) that we had previously discussed in terms of “locality and identity,” and with sociological and psychosocial factors of development (cf. Fig. 12).

Students added their own personal examples of rituals, relationships and restriction, cultural variations and differences in rituals like the high school prom (Ballagás), some historical events that determined social processes for generations (the Cold war), when Hungarian (and some Central Europeans) were not allowed to go abroad as Russia aggressively occupied their territories and restricted the freedom of their movement to the extent that the people were not allowed to travel outside the country (also relevant today in terms of the Ukrainian–Russian occupation, which is highly similar to what happened in Hungary during the Soviet occupation.

Several intercultural differences of these personal experiences were discussed, and instant applications of perspectives made it possible for participants to understand the feelings they evoked, the loss experienced (Restrictions), and the values at the individual level (in Relationships, Rituals). The pairs were required to sketch their Rituals, Relationships and Restrictions in their own culture group in pairs in class, and for the final event they were put into intercultural, international pairs where they had to *apply* that knowledge, mapping it first in their own cultures, and then going deeper for the international level. This also ensured that by the time the U.S. group was about to visit, they all had a “friend” from the partner university’s group.

Partners had to contact each other, find time outside of class to meet, and collaborate for their discussion of the locality-identity matrix given by the 3R’s. Besides giving an overview of their own locality-identity issues, they also had to confront it with their partners. Beyond specifying their own matrix, students had to reflect on their partner’s matrix from the other culture. This ensured a deep, vivid, and unique intercultural discussion of cultural values.

**Fig. 13:**
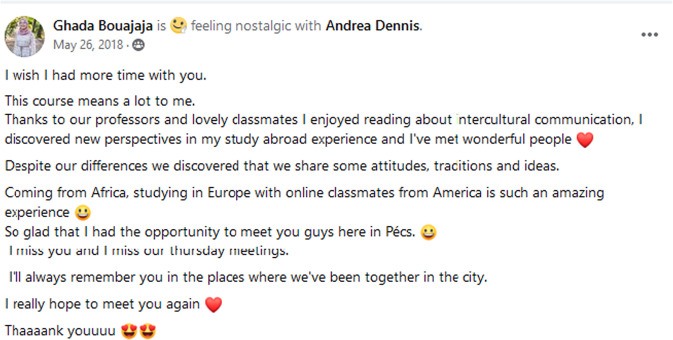
Impressions about the unique aspects of the international online joint course (video: https://www.facebook.com/petra.mieth.9/videos/1963123870418433/).

All students enjoyed the unusually open and harmonious atmosphere and the novel setting of the online joint course so much that they were all eager to participate and very enthusiastic to get answers to some culture-specific or delicate questions on the spot. One student shared her impressions in the study group about the unique aspect of the course at all levels: in terms of educational and personal experience and in terms of mind-opening adventure (Fig. 13).

### New skills yielded by the novel methodology in online academic discourse

We highlighted the new challenges in today’s society in the twenty-first century at the beginning of this article; now we would like to call the attention to the skills of *adaptability*: that proved to be crucial in terms of this new type of online joint course methodology as well. This crucial ability ensures that we can adapt to different cultures, contexts and circumstances. One of the greatest outcomes of the course is personal development in this skill.

*Versatility* of thought supports flexibility in action and thus improves intercultural communication (ICC) and performance at a global scale. In higher education we have a lot of options to include practical know-how, not only theoretical knowledge. Learning skills and know-how channel into intercultural contexts experienced through ICC. The course also serves as a platform for *socialization* in an intercultural context as it allowed instantaneous reflections on intercultural impressions about the social science topic at hand. For the U.S. students, perhaps the most important thing for improving adaptability was the visit to Hungary where they conducted their fieldwork in a very different social context.

Adaptability was also present in the *flexibility* of the professors and students: we had several pitfalls and funny events, like differences in the daylight savings management and handling time zone differences in coordinating semester schedules and university organizational rules.

All in all, this new methodology ensures personality development at many levels for all participants and is a window on the world of entire social processes at a global scale, yet in our own classrooms and communities.

## Conclusions

The collaborative online educational methodology offers exceptional possibilities for participants to experience cultural immersion in their own classroom, and to discuss the relevant questions at hand from very different perspectives. The reflections on the topics in question are instantaneous, thus facilitating the integration of perspectives, which gradually and naturally makes participants more open-minded.

Given that the students who participated represented a variety of cultures from around the globe, this made it possible to discuss one topic in a perspective ranging from collectivistic to individualistic cultures, along a continuum. This unique trait enabled participants and professors alike to gain new insight into different cultural interpretations and to understand societal challenges and obstacles to social change. As Professor Podeschi noted “Pécs brings the world to us”—as the joint course with the University of Pécs actually enabled all the participants, in all cultures and from different parts of the world, to discuss questions at a global scale, truly encompassing several cultures and continents, and thus different psychosocial perspectives.

**Fig. 14:**
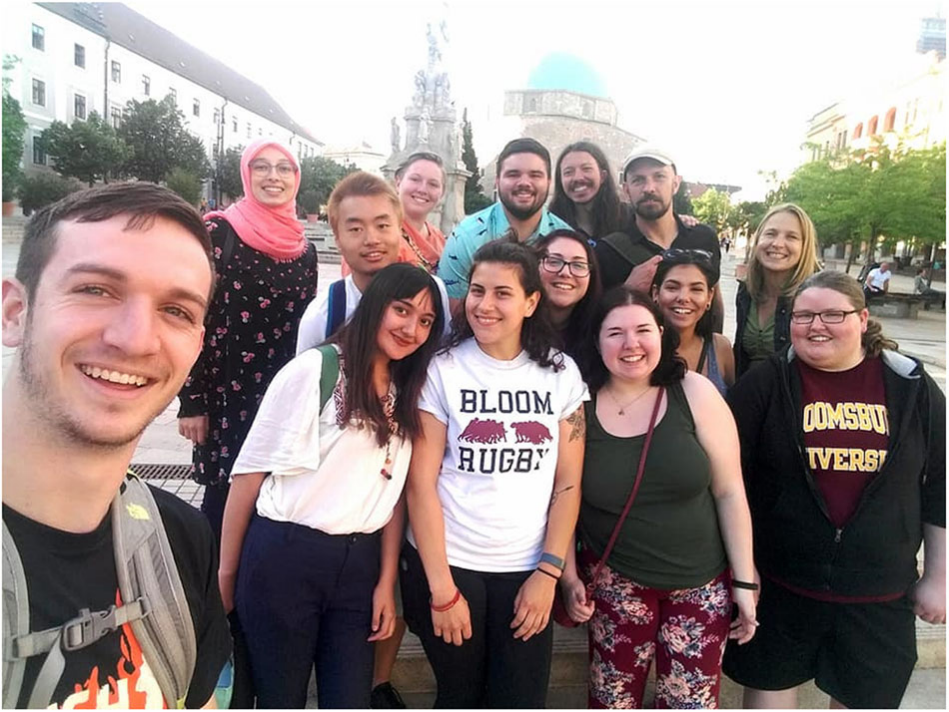
Course participants and professors in front of the mosque, a symbol of multiculturalism in the city of Pécs.

The experience ensured the formation of many long-term friendships and professional connections. This online methodology enabled students to gain first-hand experience with other cultures and societies and with communication in these cultures and societies. This is so much more valuable and much more important for students of the twenty-first century than getting ready-baked perspectives from coursebooks, through somebody’s cultural lenses and ready-made interpretations. Participants in this novel platform actually have the possibility to actively take part in and experience other cultures, but in their own city and classroom (Fig. 14). It was an experience that was truly memorable and effective in shaping the minds of new generations in education, and in discourse inside and outside the Academia.

## Data Availability

The datasets generated during and/or analyzed during the current study are available from the corresponding author on reasonable request.
